# The Role Played by Computation in Understanding Hard Materials

**DOI:** 10.3390/ma4061104

**Published:** 2011-06-14

**Authors:** John Edward Lowther

**Affiliations:** DST/NRF Centre of Excellence in Strong Materials and School of Physics, University of the Witwatersrand, Johannesburg 2094, South Africa; E-Mail: john.lowther@wits.ac.za; Tel.: +27-11-717-6829; Fax: +27-11-717-6879

**Keywords:** boride, nitride, *ab-initio*, crystal structure, elastic constants

## Abstract

In the last decade, computation has played a valuable role in the understanding of materials. Hard materials, in particular, are only part of the application. Although materials involving B, C, N or O remain the most valued atomic component of hard materials, with diamond retaining its distinct superiority as the hardest, other materials involving a wide variety of metals are proving important. In the present work the importance of both *ab-initio* approaches and molecular dynamics aspects will be discussed with application to quite different systems. On one hand, *ab-initio* methods are applied to lightweight systems and advanced nitrides. Following, the use of molecular dynamics will be considered with application to strong metals that are used for high temperature applications.

## 1. Introduction

There have been many reviews of superhardness dealing with understanding new materials [1,2,3,4,5]. Several new materials have been suggested, some synthesized and others not. The search for new materials is considerably aided through theory and especially *ab-initio* modeling procedures. Density functional theory [6,7,8] in particular has played an important role here. This “*ab-initio*” approach is applied at various levels and has been shown to lead to some significant results regarding the prediction of structural, elastic and often thermodynamic properties. There are differing opinions as to which of the approaches is best, but in general; trends are often independent of the fundamental formalism.

Properties of diamond are well known—it is the hardest material and synthesized from graphitic precursors. Cubic boron nitride is another commercially used material likewise synthesized from a similar graphitic structured material as diamond. More recent superhard materials exploiting the properties of boron, carbon and nitrogen have been examined at both theoretical [9,10,11] and experimental [12,13,14] levels. In general, the calculational insight has afforded a good interpretation of the materials synthesized although mysteries still remain. 

Quartz has many different polytypes depending upon the relative orientation of SiO_2_ tetrahdrea [15,16,17,18]. Under pressure, a superhard phase of SiO_2_ named stishovite is created, consisting of octahedral SiO_2_ units [16,19]. However, this material has not been stabilized and as such rapidly reverts back to a quartz related amorphous structure [20]. There is also a very high pressure transformation of stishovite into a CaCl_2_ form [21] with some related enhancement of elastic properties. Thereafter enhancement of the coordination has required metals which involve d states. In this way the near nine-fold coordinated TiO_2_ cotunnite phase has now been suggested to be the hardest known oxide [22]. However, synthesis of this material needs very high pressures (in excess of 50 GPa) while similar cotunnite phases of ZrO_2_ or HfO_2_ are synthesized at lower pressures between 12–18 GPa [23,24,25] and could also show superhard features.

Other hard materials that have been identified from computation [26,27] have been based upon some heavy metals such as Os, Re or Rh [28] forming nitrides [29]. The noble metals in themselves are also proving quite important in the formation of hard superalloys. Nickel-based superalloys are highly efficient materials for engines because of interactions between component phases. Exceptional high-temperature properties of the nickel superalloys have been attributed to its microstructure. Despite the success of such alloys, they are presently at the limit of their high-temperature and here the incorporation of noble metals has been suggested [30].

The purpose of this paper is to consider some examples of novel materials where computation has provided some insight and possibly even prediction. Computation aspects will extend from the “*ab-initio*” level to the more empirical aspect as used in molecular dynamics.

## 2. Computational Approaches

There are now various very efficient computer algorithms available that can evaluate the total energy of a system using density functional approaches. Choice is often associated with available resources and notably computer power. This paper does not wish to cover the fundamental aspects of the density functional approach other than to remind the reader that effectively the theory is still under development and that many problems, e.g., the application to excited states, energy gaps, *etc*., are still being studied. However, so saying for ground state properties of a particular ensemble of atoms, two choices of density functional theory are especially significant. These are the local density approximation (LDA) [31] and the Generalized Gradient Approximation (GGA) [32]. Several computations—namely VASP [33], SIESTA [34], ESPRESSO [35] among others—are proving useful. One useful aspect of the codes is the use of pseudopotentials [36,37] and the plane wave approach [38]. 

Other approaches that involve molecular dynamics can handle far larger systems of atoms than the *ab-initio* approaches. However, this comes at some cost, namely in the description of the inter-atomic potentials and these tend to take some kind of analytical form. Of the various codes that can implement such techniques, DLPOLY [39] and GULP [40] are examples. Various thermodynamic conditions can be imposed in the simulations of extended systems and usually many thousands of atoms can be involved.

Using such approaches it is possible to deduce a total electronic energy of the system, *E_tot_*, and from variation of this with a unit cell distortion the elastic constants (*c_ij_*) and thereafter an effective Voigt isotropic bulk (*B_v_*) and shear elastic moduli (*G_v_*) through an expression of the form:

$$B_{v}=\frac{1}{9}[c_{11}+c_{22}+c_{33}+2(c_{12}+c_{13}+c_{23})]$$

$$G_{v}=\frac{1}{15}[c_{11}+c_{22}+c_{33}+3(c_{44}+c_{55}+c_{66})-c_{12}-c_{13}-c_{23}]$$

It is often thought the both moduli—or better the shear—relate to hardness but this has to be treated carefully especially when anisotropic materials are concerned. A mechanical stability can also be established for a specified system—where the shape of the system will sustain an external force. In the case of a simple cubic system where there are only three elastic constants, namely *c_11_*, *c_12_* and *c_33_*, the stability criteria is [41]:

(*c_11_ − c_12_*) > 0; *c_44_* > 0; (*c_11_ + 2c_12_*) > 0

The requirement of mechanical stability for orthorhombic crystals with nine elastic constants are as follows:

$$(c_{11}+c_{22}-2c_{12})>0,$$

$$(c_{11}+c_{33}-2c_{13})>0,$$

$$(c_{22}+c_{33}-2c_{23})>0,$$

$$c_{11}>0,c_{22}>0,c_{33}>0,c_{44}>0,c_{55}>0,c_{66}>0\;$$

$$(c_{11}+c_{22}+c_{33}+2c_{12}+2c_{13}+2c_{23})>0.$$

Theory can only give an approximate estimate of the transition pressures between phases as the precise mechanism of the transition often requires some subtle dynamic processes. However, the relative energy between the phases is an important indicator with very small differences implying low transition pressures needed between different phases. In fact, the region where one material transforms to another can involve the existence of a highly metastable system where very little is understood. 

It has often been noted that although the GGA gives a good description of the lattice geometry, LDA may be better for elastic constants and moduli. However, use of the fundamental approach gives results that still need caution in a predictive modeling context. 

## 3. Boron Carbon Nitrogen Structures

There are many boron related structures composed of elemental boron icosahedra formed from 12 boron atoms [42,43] and similar boron structures are found in important hard materials like B_4_C, B_6_O or MgAlB_4_ *etc*. [44]. Some of the latter materials are established for application or are under research for further improvement [45]. Other structures based on B–C or B–C–N are under intense investigation for their potential to rival the properties of diamond because of chemical differences and to produce new application. Of importance to the synthesis of these materials are the precursor phases. As with diamond, graphitic structures are considered to be the most likely possibilities here. However, in this respect, it is noted that there are differences in the relative energetics of the diamond-graphite systems as compared with the cubic BN-hexagonal BN system. In the case of the diamond-graphite system, the energy of diamond lies above that of graphite [46] whereas for the c-BN-hBN system it is the reverse [47,48]. 

### 3.1. Graphitic Precursor Structures

**Figure 1:**
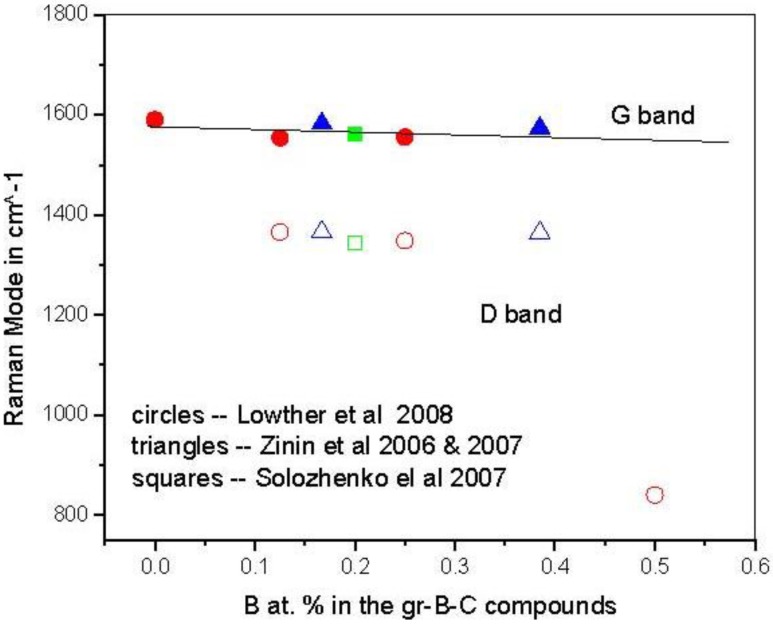
Comparison of vibration energies of the G and D Raman bands in various B–C graphitic structures. Circles are calculations [49], triangles from Zinin *et al.* [50,51] and squares from Solozhenko *et al*. [52].

**Figure 2:**
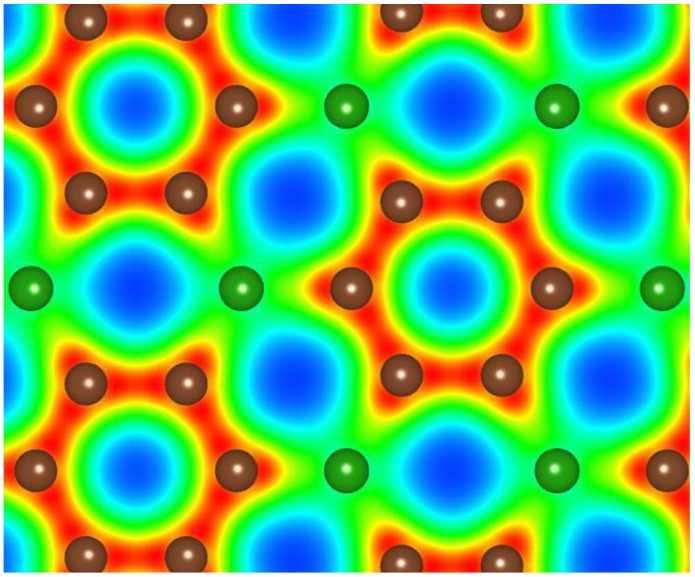
Calculated electronic structure of g-BC_3_ (adapted from [49]).

Precursor phases to either a B–C or a B–C–N related structure are graphitic [13]. In the case of the B–C graphitic system, Raman measurements of BC_n_ graphitic structures have now been obtained and the frequencies are somewhat consistent with theory [49] as shown in Figure 1. The theory assumed flat graphitic related BC_n_ sheets as indicated in Figure 2, but it likely that these will slightly buckle and this could account for the very marginal differences between theory and experiment. 

**Figure 3:**
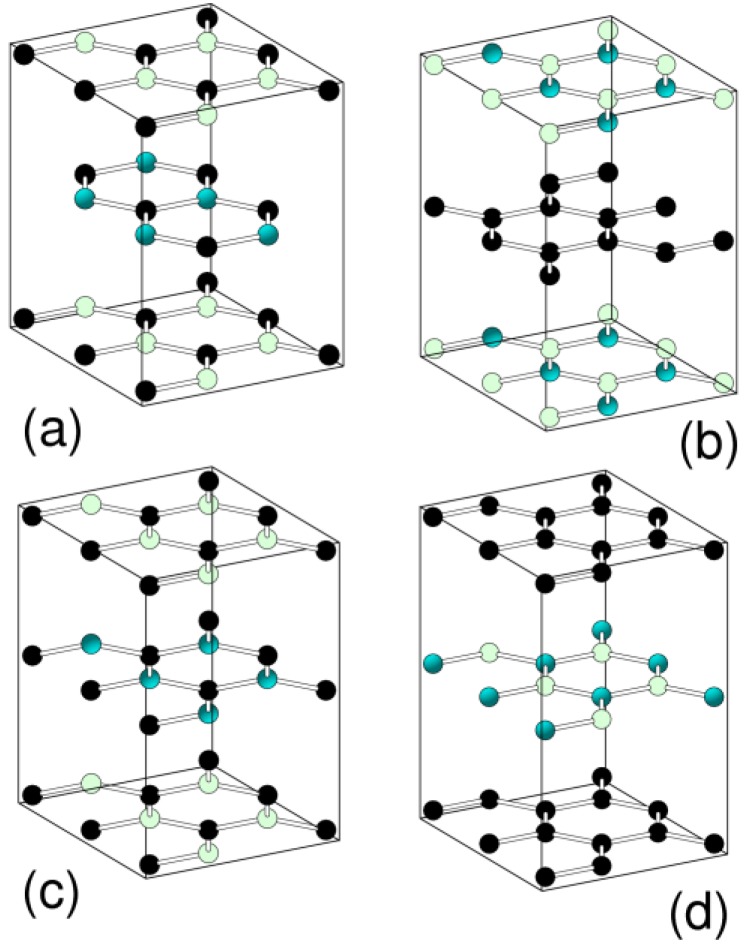
Various graphitic structures considered for hexagonal BC_2_N. Structures (**a**) and (**c**) follow stacking as for graphite, structures (**b**) and (**d**) as for h-BN. The shading of spheres is white B, black C and grey N.

On the contrary, with B-C-N graphic phases, there is some suggestion that there is a separation of the sheets into C and B-N structures [53]. Calculations have been performed on the graphitic structures shown in Figure 3.

From Table 1, it is clear that the h-BC_2_N structures (b) and (d) have the lowest energy, consistent with a sheet phase separation in this material. This phase separation could be somewhat disappointing from the superhard synthesis of a crystalline diamond-like superhard phase of BC_2_N. In fact, suggested crystalline structures of diamond-like BC_2_N have been studied using *ab-initio* approaches [54] with several forms having large elastic moduli again consistent to that being found. But the superhardness of such a material is likely to relate mainly to the number of C-C bonds in the material. Calculations have also suggested that large amounts of B in diamond would lead to graphitization [55].

**Table 1:** Cell structure and calculated total energy of hexagonal BC_2_N structures. Experimental values from [53].

	a (*A*)	c (*A*)	*E_tot_* (eV/atom)
h-BC_2_N (a)	2.50	5.74	−8.456
h-BC_2_N (b)	2.47	6.41	−9.880
h-BC_2_N (c)	2.50	5.89	−8.440
h-BC_2_N (d)	2.47	6.82	−9.868
Expt.	2.42	7.25	

The transition pressure from the various graphitic phases of both BC_n_ and BC_2_N structures appear to be quite similar around P = 18 GPa and around 2000 K. Although the structures are metastable relative to diamond/graphite or phases of BN, this is a difficulty that will be overcome [56]. 

### 3.2. Superhard Structures

There are many attempts to understand the nature of the superhard structures of the B-C-N system but most are based upon the structure of diamond. Simple modification in the case of either a BC_n_ [10] or BC_2_N [54] structure has been used. Relative to diamond and graphite, all of these structures are metastable from the point of view of the relative energies and this will be a problem to overcome in a satisfactory synthesis. The essential conclusion that is emerging in the case of the superhard BC structures is that the number of C-C bonds will dictate the ultimate strength of the material [57]. Although there is still some uncertainty as to how much boron can actually be incorporated into a diamond structure, recent calculations suggest that a very heavily B-doped material will easily graphitize [55]. 

The situation for a material like BC_2_N is not as straightforward as the BC_n_ system obviously because of the presence of both B-N and C-C bonds. Although various structures have been speculated for this material [58,59,60,61,62,63,64], as yet a precise structure is not determined. However, there is some indication that the relative energies of these structures are quite close.

As with diamond-graphite and cBN–h–BN, it is interesting to compare similar phases of BC_3_ and BC_2_N. Results of LDA calculations are shown in Table 2—the results suggest that N concentration dramatically affects the relative energy and may possibly affect the transition pressure.

**Table 2:** Local density approximation (LDA) calculated relative energies of precursor graphitic and super-hard diamond-like structures. A positive value indicates that the precursor phase is higher in energy that the diamond-like phase. The estimated calculational uncertainty is 10 meV/atom.

Stoichiometry	hexagonal phase	Diamond-like phase	Energy difference (eV/atom)
BC_2_N	h-BC_2_N	BC_2_N	−0.397
BC_3_	BC_3_	BC_3_	+0.058
C	graphite	diamond	−0.003
BN	h-BN	c-BN	+0.056

## 4. Advanced Nitrides

Binary nitrides of transition metals constitute a diverse class of materials with technological and fundamental importance because of their strength and durability as well as their optical, electronic and magnetic properties [65,66]. Oxidation states of the binary nitrides of transition metals are limited by the metal even if the number of available valence electrons is high [67]. For these reasons three metal dinitrides—PtN_2_ [68], InN_2_ and OsN_2_ [69]—have successfully been synthesized under extreme conditions of pressure and temperature. There have been several studies of the crystal structure of these nitrides [70,71,72], some pointing to an enhanced bulk modulus of the nitride over the metal with a rock-salt structure being the most stable structure [72] or a pyrite structure [73] and this arises due to strong hybridization between the metal d and N 2p states [74]. 

More recently, nitrides of tantalum have become a rapidly growing field of interest. The binary Ta-N system display rich compounds with well defined variable stoichiometry [67,75]. This crystal chemistry ranges from Ta_3_N_6_, Ta_4_N_5_ up to Ta_3_N_5_—three polymorphs of the mononitride TaN and two phases of Ta_2_N. High pressure δ-*TaN* having a NaCl structure and orthorhombic Ta_3_N_5_ have outstanding properties among the Ta-N system and are said to be superconducting. 

**Figure 4:**
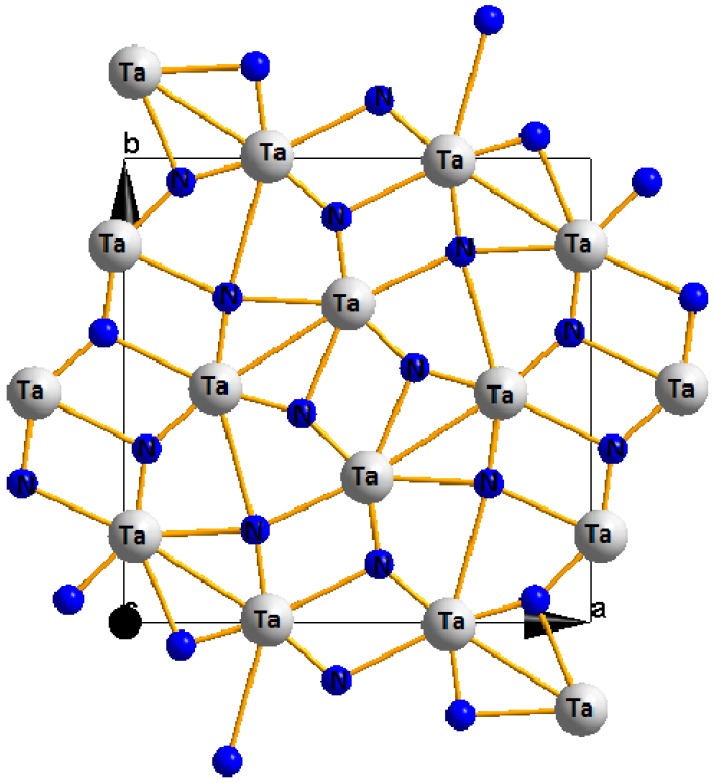
Crystal structure of orthrhombic Ta_2_N_3_.

Very recently, Zerr *et al*. [65] synthesized the novel tantalum nitride having an orthorhombic U_2_S_3_ structure (space group *Pbnm*) at high pressure and temperature conditions with the aim of obtaining the high pressure phase. The structure is shown in Figure 4.

From the experimental findings of Zerr *et*. *al*., the orthorhombic Ta_2_N_3_ displays high hardness and a unique texture and this makes it a potential superhard material for industrial applications.

Recent compuational studies using the GGA approach [76], however, have suggested the Ta_2_N_3_ is unstable as one of the elastic moduli (c_66_) is less tha zero and contravenes the Born criteria for orthrhombic systems. This is so despite the other moduli—buk or shear are quite large and indicators of a superhard material. However, more recent calculations using LDA are not consistent with this interpretation and, in fact, predict all elastic constants that are consistent with the Born criteria, thus indicating the stability of the orthorhombic structure of Ta_2_N_3_.

## 5. Strong Metallic Alloys

Nickel-based superalloys (NBSAs) are presently used for engines primarily because of highly efficient material properties including resistance to oxidation and corrosion, elevated temperature strength, relatively low density and high melting points. Unlike most intermetallics, NBSAs also have excellent ductility both at room and elevated temperatures, even though they are not often considered as being hard materials at room temperature. NBSAs are either solid solution or precipitation strengthened. The exceptional high-temperature properties of NBSAs have been attributed to an interplay between two microstructures—a f.c.c phase and a more elaborate one. This latter type of micro-structure has been found to demonstrate exceptional structural stability and best characteristics of high temperature strength, resulting in their usage in aircraft engines. Despite the success of NBSAs, they are presently at the limit of their high-temperature capability [77]. Due to increased demand for system efficiency, other alloys capable of higher temperature operation are required. Advantages gained with higher system efficiency are high throughput and less environmental pollution and alloys based on the noble metals are therefore currently being investigated as material for high temperature alloy design [78,79]. Although, noble metals offer the hope of developing exceptional alloys capable of high temperature applications, considerable effort is also required to understand the fundamental factors that control their mechanical properties as they also exhibit a variety of phases [80].

**Figure 5:**
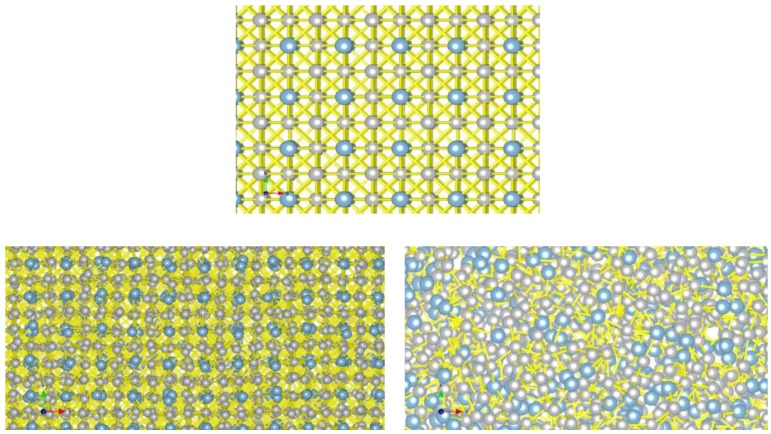
Molecular Dynamics results for the melting of Ni_3_Al [83]. Top: T = 0 K; bottom left T = 1000 K and bottom right T = 2000 K.

Ni_3_Al has proven an interesting material for both *ab-initio* [81,82] and molecular dynamic simulation [83,84]. Sutton-Chen potentials [85] seem quite good in simulating this phase with calculations of the melting temperatures being in quite good agreement with experimental. Such calculations show the distribution of atoms at various temperatures—a typical result is shown in Figure 5 for the cubic structure of Ni_3_Al. As can be seen, initially the cubic lattice has only slightly deformed but at very higher temperatures there is no sign of a cubic lattice, showing that the material essentially has melted. A more quantitative analysis of the behavior is based upon the behavior of the diffusion coefficient of the various component atoms which increases dramatically at the melting temperature. 

The use of platinum in a Pt_3_Al alloy has been considered as a possible alternative to Ni_3_Al as a high temperature strong material but is currently hampered by its weight and high cost. However these factors are expected to be counterbalanced by platinum’s exceptional chemical stability, oxidation resistance, ductility, thermal-shock resistance, and electrical or thermal conductivity, which makes it desirable for the design of next generation of super-alloys [86,87]. 

## 6. Conclusions

There are two regions of computational materials modeling that are proving useful in not only understanding materials but also, and more important, predicting new ones. The *ab-initio* approach is quite reliable for ground state properties—elastic constants and lattice geometries—albeit the various density functional approaches do not agree even though similar trends are suggested. On the other hand, very large systems can be examined using various forms of rigid potential molecular dynamics. At this stage in the theoretical development, it is not really clear how the *ab-initio* methods will be able to be extended into the same region as the rigid potential molecular dynamics but despite this rather major shortcoming there have been many achievements in materials modeling and this will continue.
